# Polychaete dataset of the southwestern Gulf of Mexico including: taxonomic checklist, abundance, bathymetric distribution, functional diversity, geographic location, and sampling sites depth

**DOI:** 10.1016/j.dib.2022.108370

**Published:** 2022-06-13

**Authors:** Octavio Quintanar-Retama, Ana Rosa Vázquez-Bader, Adolfo Gracia

**Affiliations:** a Universidad Nacional Autónoma de México (UNAM), Instituto de Ciencias del Mar y Limnología, Unidad Académica Ecología y Biodiversidad Acuática, A.P. 70-305 Ciudad Universitaria 04510 México, CDMX, México; b Posgrado en Ciencias Biológicas, Unidad de Posgrado, Edificio D 1° Piso, Circuito de Posgrados, Ciudad Universitaria, Alcaldía Coyoacán, C.P. 04510, Cd. Mx., México

**Keywords:** Polychaeta, Deep-sea, Gulf of Mexico, taxonomic checklist, abundance, functional diversity

## Abstract

A taxonomic list of 69 genera belonging to 33 families of the Polychaeta class (Annelida) collected in 54 deepwater sites of the southwestern Gulf of Mexico is presented. Abundance data of these 69 genera is also included. A dataset of geographical location and depth of sampling sites is given. Graphs of depth related community functional diversity variation are shown. The biological material was obtained from sediment samples collected aboard the Justo Sierra Oceanographic Vessel of the National Autonomous University of Mexico using a Reineck-type box corer with an effective area of 0.16 m^2^. In each core a subsample of 0.08 m^2^ and 13 cm deep was taken and washed through a 500-micron sieve with filtered seawater. Abundances were standardized to individuals per square meter. The average abundance contribution percentage graphs were done calculating the average standardized abundance of each guild and the contribution percentage of each one to the four depth categories established: Upper bathyal zone (UBZ); middle bathyal zone (MBZ); lower bathyal zone (LBZ) and abyssal zone (ABYZ). These data could be useful for comparative purposes with new data of polychaete communities in the same area or another region.

## Specifications Table

**Table:** 

Subject	Biological Sciences (Biodiversity)
Specific subject area	This dataset provides information about the polychaete biodiversity and ecology of the southwestern deep sea of the Gulf of Mexico.
Type of data	Table Figure
How the data were acquired	The biological material was obtained in two cruises carried out on board the Oceanographic Vessel Justo Sierra of the National Autonomous University of Mexico. The samples were collected using a Reineck-type box corer with an effective area of 0.16 m^2^. Once the corer was on deck, subsamples of 0.08 m^2^ were taken, which were washed on board through a 500-micrometer mesh sieve with previously filtered seawater. The sieved material was fixed with a mixture of seawater and 8% formalin. In the laboratory, specimens were separated from the sediment using an AVEN Mighty Vue Pro 5D ESD magnifying lamp (2.25X magnification) and fine-tipped tweezers. The specimens were kept in vials with 70% alcohol. Subsequently, the polychaetes, whose preservation status allowed it, were identified at the genus level and individuals number were included in the abundance matrix. Taxonomic identification at the genus level was performed using a Zeiss Stemi 508 stereoscopic microscope (maximum magnification 50X) and a Zeiss Primo Star microscope in addition to specialized literature. The assignment to a trophic guild was after the taxonomic identification and was carried out using specialized literature. The sampling sites geographical location, and depth was registered with ship GPS, and multibeam echosounder, respectively.
Data format	Analyzed
Description of data collection	Two oceanographic cruises based on a systematic sampling design with 63 locations were conducted in the southwestern Gulf of Mexico. The first cruise was carried out on June 3- 27, 2015. Due to logistical reasons, sediment samples were only collected at 60 sites. Biological material of seventeen samples (not included in this data set) was lost before the genus-level identification of the polychaetes was achieved. Polychaetes were not obtained in two locations, besides three samples with organisms no identified at the genus level were not included in the abundance matrix, resulting in 38 sites in the first cruise. The second cruise was carried out on August 31- September 20, 2016. Sediment samples were successful at 60 sites. Polychaetes could not be identified at the genus level in nine locations and were not collected in five sites, so they were not included in the analysis. This resulted in 46 sites with polychaetes identified at the genus level in the second cruise. A common abundance matrix for both cruises was elaborated. In those sites where it was possible to register and identify polychaetes at genus level in both cruises, the organism numbers were summed and standardized to individuals per square meter. In those locations with a single record of any of the cruises, the data was just added to the abundance matrix. In this way a total of 54 sites with polychaetes identified at the genus level were recorded for both cruises. The sites were renamed from one to 54 consecutively. The geographical location and depth were registered with ship GPS and multibeam echosounder EM302, respectively, at the time the corer reached bottom.
Data source location	• Instituto de Ciencias del Mar y Limnología, Universidad Nacional Autónoma de México • Southern Gulf of Mexico • México Site Longitude W Latitude N • 1 93.4971 18.9898 • 2 94.0183 19.0093 • 3 94.5077 18.7427 • 4 94.9991 18.8284 • 5 95.5017 19.0283 • 6 94.9799 19.1478 • 7 93.516 19.5089 • 8 94.0166 19.5116 • 9 94.5069 19.5128 • 10 94.9963 19.4969 • 11 95.5051 19.4992 • 12 96.0114 19.5083 • 13 96.0051 20.0064 • 14 95.5155 20.0012 • 15 94.5154 19.9996 • 16 94.0306 20.0211 • 17 93.0128 20.0034 • 18 92.5696 20.5033 • 19 93.5075 20.4969 • 20 94.5185 20.4991 • 21 95.0113 20.4901 • 22 95.5111 20.4937 • 23 96.0123 20.5099 • 24 96.5006 20.403 • 25 96.5102 20.5161 • 26 96.0053 21.0042 • 27 95.0197 20.985 • 28 94.4945 20.9801 • 29 93.9818 21.0018 • 30 93.0018 21.0129 • 31 92.6268 21.5039 • 32 93.5061 21.5138 • 33 94.5227 21.4989 • 34 95.0251 21.4773 • 35 96.0152 21.5038 • 36 96.512 21.5155 • 37 96.512 22.0091 • 38 96.0245 22.018 • 39 95.0248 21.9997 • 40 94.5235 22.0033 • 41 94.0463 21.9861 • 42 93.5309 22.4993 • 43 94.0283 22.5098 • 44 94.4936 22.5017 • 45 94.983 22.4916 • 46 95.5217 22.4997 • 47 96.01 22.5054 • 48 96.5036 22.5009 • 49 96.0146 23.0109 • 50 95.5151 22.9864 • 51 95.0153 22.992 • 52 94.5171 23.0055 • 53 94.0106 23.0215 • 54 93.0106 22.9984
Data accessibility	Mendeley data Data identification number: https://doi.org/10.17632/bz8dr9cjpt.2 Direct URL to data: https://data.mendeley.com/datasets/bz8dr9cjpt/2
Related research article	***For an article which has been accepted and is in press:*** Quintanar-Retama, O., Armenteros, M., Gracia, A., Diversity and distribution patterns of macrofauna polychaetes (Annelida) in deep waters of the Southwestern Gulf of Mexico, Deep-Sea Research Part I, 181 (2022), doi: https://doi.org/10.1016/j.dsr.2022.103699. [1].

## Value of the Data

• These data are new, unique and may serve as a basis for further studies in the Gulf of Mexico. Diversity and ecological studies on a poorly studied area like this is of outmost importance and usefulness for future deep-sea research in the region. Data is of great importance since polychaetes, functional traits, and macrofauna in general, in this deep sea area is scarce and knowledge is necessary for understanding the deep sea ecosystem.
• Researchers, students, and stakeholders interested in the ecology, biodiversity, and resilience of deep-sea benthic communities of the Gulf of Mexico will have the opportunity to use this data as baseline for planning further studies and assessing potential impacts due to human actions. Even more, this data can be useful to enhance knowledge and function of deep-sea communities in other parts of the world
• These data can complement larger data sets on the same taxon (Polychaeta) or on other taxa of the deep sea macrofauna in the Gulf of Mexico or elsewhere. It can be used on studies to relate with other fauna categories like meiofauna or megafauna of the region. It could be useful to make comparisons with scientific research in other areas and integrate a global knowledge of the deep sea.

## 1 Data Description

**Fig. 1:**
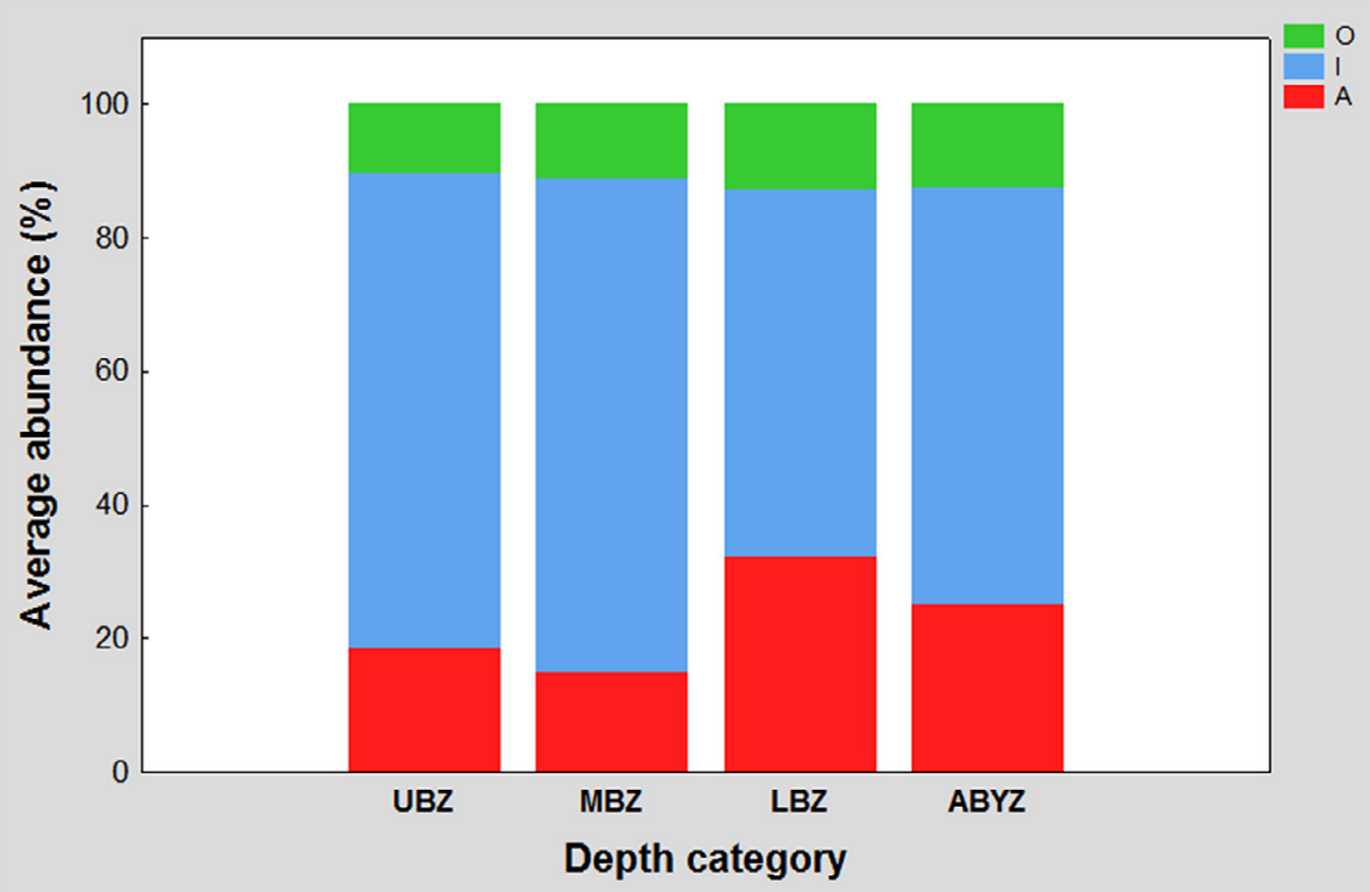
General feeding guilds contribution percentage to average abundance in each depth category. The letters meaning in the acronym are I = microphages; A = macrophages, and O = omnivores. Upper bathyal zone (UBZ); middle bathyal zone (MBZ); lower bathyal zone (LBZ) and abyssal zone (ABYZ).

Figure 1 shows the contribution percentage of the three general feeding guilds (macrophages in red, microphages in blue, and omnivores in green) recorded in the study area for each of the established depth categories (upper bathyal, middle bathyal, lower bathyal and abyssal). The four bars represent one hundred percent of the abundance recorded in its corresponding depth category and the length of the segment of each color determines the percentage contribution of each trophic guild.

**Fig. 2:**
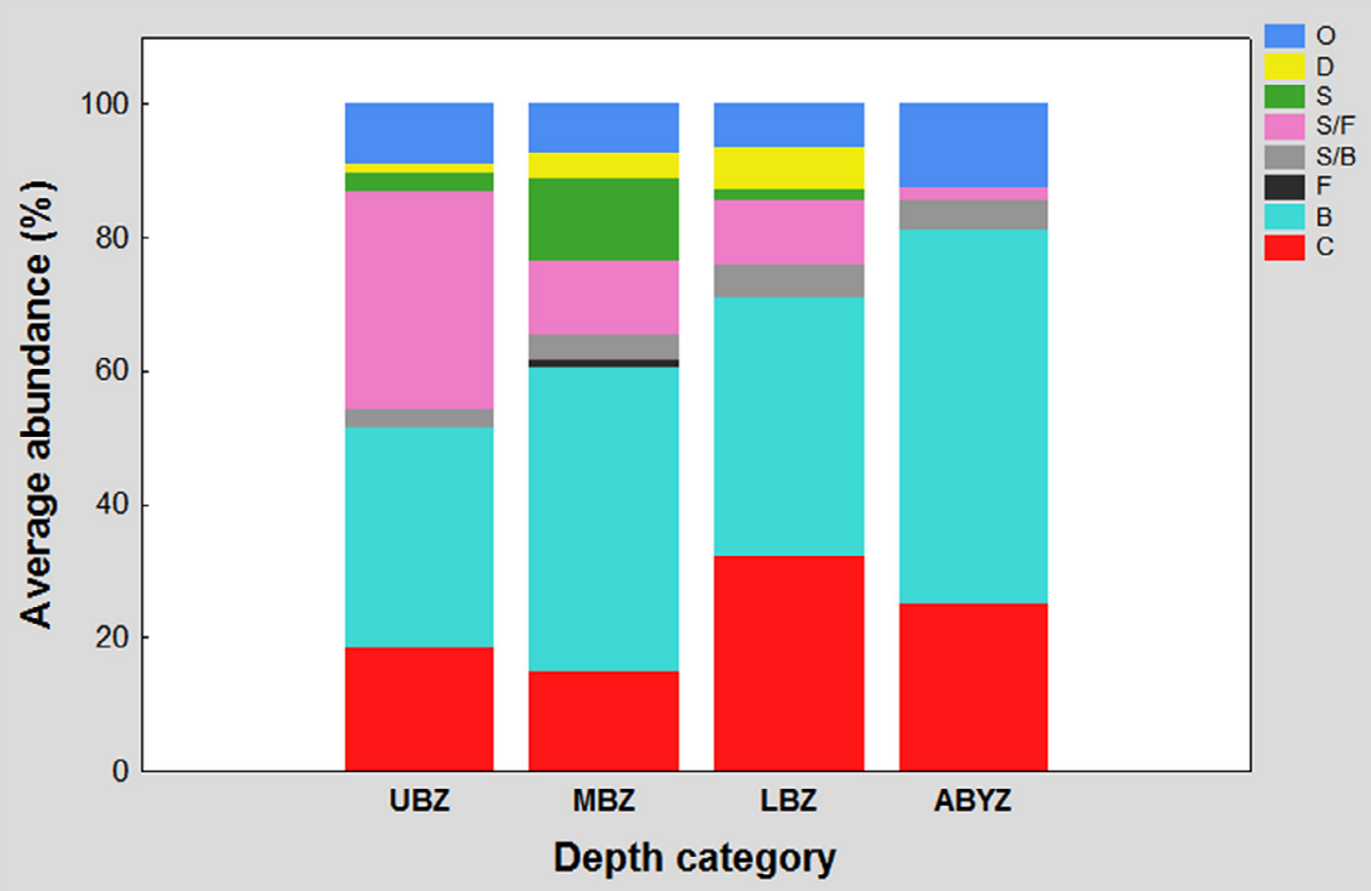
Specific feeding guilds contribution percentage to average abundance in each depth category. The letters meaning in the acronym are B = subsurface deposit feeders; S = surface deposit feeders; F = suspension feeders; O = omnivores, D = detritivores, and C = carnivores/scavengers. Upper bathyal zone (UBZ); middle bathyal zone (MBZ); lower bathyal zone (LBZ) and abyssal zone (ABYZ).

Figure 2 shows the percentage contribution of the eight specific feeding guilds (carnivores/scavengers in red; subsurface deposit feeders in aqua green; suspension feeders in black; surface deposit feeders / subsurface deposit feeders in grey; surface deposit feeders / suspension feeders in pink, surface deposit feeders in green, detritivores in yellow, and omnivores in blue) recorded in the southwestern Gulf of Mexico for each of the established depth categories (upper bathyal, middle bathyal, lower bathyal and abyssal). The four bars represents one hundred percent of the abundance recorded in its corresponding depth category and the length of the segment of each color determines the percentage contribution of each trophic guild.

This graph shows the contribution percentage of the three motility traits (discretely motile in blue, motile in red and sessile in yellow) recorded in the study area in each of the four established depth categories (upper bathyal, middle bathyal, lower bathyal, and abyssal). The four bars represent one hundred percent of the recorded abundance in its corresponding depth category and the length of the segment of each color determines the contribution percentage of each motility trait.

This file presents a taxonomic checklist of the 33 families and 69 genera of polychaetes identified at 54 deep-water sites in the southwestern Gulf of Mexico. This list also contains the name of the taxonomic authority and the year of description for each taxon.

**Dataset 1.** Polychaeta standardized abundance.

This dataset includes standardized abundance data of 69 polychaetes genera recorded at 54 deep-water sites in the southwestern Gulf of Mexico. Abundance values were standardized to individuals per square meter.

**Dataset 2.** Sampling sites geographic location and depth data

Geographic and bathymetric data set of the sampling sites. The list includes the name, geographic coordinates and depth recorded at each of the 54 sampling sites.

## 2 Experimental Design, Materials and Methods

### 2.1 Sampling and sample processing

The biological material was obtained along two cruises carried out on board the Oceanographic Vessel Justo Sierra of the National Autonomous University of Mexico. The samples were collected using a Reineck-type box corer with an effective area of 0.16 m^2^. Once the corer was on deck at each sampling site, a 0.08 m^2^ subsample was taken and washed on board through a 500-micrometer mesh sieve with previously filtered seawater. The result of this sieving was fixed with a mixture of seawater and 8% formalin. In the laboratory, the samples were washed with tap water through a 500-micrometer mesh sieve to remove the residue of the used fixative. The extraction of the polychaetes specimens was carried out placing the sediment of each sample in Petri dishes of 15 cm in diameter in small volumes until the sample was finished. To visualize the specimens, an AVEN Mighty Vue Pro 5D ESD magnifying lamp (2.25X magnification) was used. The specimens were separated using fine-tipped tweezers and placed in vials with 70% alcohol.

### 2.2 Taxonomic checklist

The taxonomic identification was done observing the specimens in Petri dishes of 5 cm in diameter with water under a Zeiss Stemi 508 stereoscopic microscope (maximum magnification 50x). When it was necessary, the specimens (or a dissected portion) were mounted on a slide with a drop of a 70% alcohol-glycerol mixture and a coverslip and were observed with a Zeiss Primo Star optic microscope. Some specimens were temporary stained with Methylene Blue or Shirlastain-A to highlight structures of taxonomic importance. General [2], [3], [4] and specialized literature [5], [6], [7], [8] was used. The validation of the names of families, genera, and taxonomic authorities, as well as the year of description of each taxon, was carried out using the WoRMS match taxa tool [9].

### 2.3 Abundance Matrix

In the original study design, 63 sampling sites were considered, however, for logistical reasons, sediment samples were only collected at 60 sites during each of the two oceanographic cruises that constitute this data set. Polychaetes identified at the genus level were collected at 38 sampling sites in SOGOM 1 and 46 in SOGOM 2. The abundance matrix was constructed adding the numbers recorded in both cruises. Thirty common sites in both cruises registered polychaetes identified at genus level. Twenty-four locations only presented polychaetes identified at genus level in one of the two cruises (8 in SOGOM 1 and 16 in SOGOM 2). This made a total of 54 sites with polychaetes identified at genus level. The sites were renamed in a scale order from one to 54 consecutively. The standardization of abundance (individuals per square meter) was done based on the number of each polychaete genus in each site. Genus number of a single cruise location was divided by 0.08, whereas data of the two cruises were added and the result was divided by 0.16.

### 2.4 Depth Categories

Four depth categories were determined according to the literature [10,11], and bathymetry of the Gulf of Mexico. These categories were: upper bathyal zone (185 -1000 m); middle bathyal zone (1001 -2000 m); lower bathyal zone (2001 -3000 m), and abyssal zone (3000 – 3760 m). The depth of each sampling site was recorded using the ship multibeam echosounder EM302, and the geographical location with the ship GPS at the time the corer got to the bottom. The depth of the sites with a single fauna record in one of the two cruises was the recorded in the data sampling sites set. An average depth was calculated for sites with data of the two cruises. Each of the 54 sites was assigned to one of the four established depth categories. Thus, 9 sites were classified into the upper bathyal zone, 11 sites in the middle bathyal zone, 16 sites in the lower bathyal zone and 18 sites in the abyssal one, Table 1.

**Table 1:** Taxonomic list of genera and families identified in the deep sea from the southwestern Gulf of México.

Phylum Annelida Lamarck, 1802
Class Polychaeta Grube, 1850
Subclass Errantia Audouin & H Milne Edwards, 1832
Order Amphinomida
Family Amphinomidae Lamarck, 1818
Genus *Paramphinome* M. Sars in G. Sars, 1872
Order Eunicida
Family Lumbrineridae Schmarda, 1861
Genus *Abyssoninoe* Orensanz, 1990
Genus *Augeneria* Monro, 1930
Genus *Lumbrinerides* Orensanz, 1973
Genus *Lumbrineris* Blainville, 1828
Family Onuphidae Kinberg, 1865
Genus *Paradiopatra* Ehlers, 1887
Order Phyllodocida Dales, 1962
Family Glyceridae Grube, 1850
Genus *Glycera* Grube, 1850
Family Goniadidae Kinberg, 1866
Genus *Goniada* Audouin & H Milne Edwards, 1833
Genus *Goniadides* Hartmann-Schröder, 1960
Genus *Progoniada* Hartman, 1965
Family Hesionidae Grube, 1850
Genus *Hesiocaeca* Hartman, 1965
Genus *Syllidia* Quatrefages, 1865
Family *Nephtyidae* Grube, 1850
Genus *Aglaophamus* Kinberg, 1866
Genus *Nephtys* Cuvier, 1817
Family Nereididae Blainville, 1818
Genus *Ceratocephale* Malmgren, 1867
Family Paralacydoniidae Pettibone, 1963
Genus *Paralacydonia* Fauvel, 1913
Family Phyllodocidae Örsted, 1843
Genus *Eteone* Savigny, 1822
Family Pilargidae Saint-Joseph, 1899
Genus *Ancistrosyllis* McIntosh, 1878
Genus *Litocorsa* Pearson, 1970
Genus *Sigambra* Müller, 1858
Family Sigalionidae Kinberg, 1856
Genus *Pholoides* Pruvot, 1895
Family Syllidae Grube, 1850
Genus *Exogone* Örsted, 1845
Genus *Pionosyllis* Malmgren, 1867
Subclass Sedentaria Lamarck, 1850
Infraclass Canalipalpata Rouse & Fauchald, 1997
Order Sabellida Levinsen, 1883
Family Sabellidae Latreille, 1825
Genus *Euchone* Malmgren, 1866
Order Spionida Rouse & Fauchald, 1997
Family Spionidae Grube, 1850
Genus *Aonides* Claparède, 1864
Genus *Dispio* Hartman, 1951
Genus *Laonice* Malmgren, 1867
Genus *Malacoceros* Quatrefages, 1843
Genus *Paraprionospio* Caullery, 1914
Genus *Prionospio* Malmgren, 1867
Genus *Spiophanes* Grube, 1860
Family Longosomatidae Hartman, 1944
Genus *Heterospio* Ehlers, 1874
Family Poecilochaetidae Hannerz, 1956
Genus *Poecilochaetus* Claparède in Ehlers, 1875
Family Trochochaetidae Pettibone, 1963
Genus *Trochochaeta* Levinsen, 1884
Order Terebellida Rouse & Fauchald, 1997
Family Ampharetidae Malmgren, 1866
Genus *Ampharete* Malmgren, 1866
Genus *Amphicteis* Grube, 1850
Genus *Auchenoplax* Ehlers, 1887
Genus *Eclysippe* Eliason, 1955
Family Cirratulidae Ryckholt, 1851
Genus *Aphelochaeta* Blake, 1991
Genus *Chaetozone* Malmgren, 1867
Genus *Kirkegaardia* Blake, 2016
Family Fauveliopsidae Hartman, 1971
Genus *Laubieriopsis* Petersen, 2000
Family Flabelligeridae de Saint-Joseph, 1894
Genus *Bradabyssa* Hartman, 1967
Genus *Diplocirrus* Haase, 1915
Family Sternaspidae Carus, 1863
Genus *Caulleryaspis* Sendall & Salazar-Vallejo, 2013
Genus *Sternaspis* Otto, 1820
Family Trichobranchidae Malmgren, 1866
Genus *Terebellides* Sars, 1835
Infraclass Scolecida Rouse & Fauchald, 2001
Family Capitellidae Grube, 1862
Genus *Mediomastus* Hartman, 1944
Genus *Neoheteromastus* Hartman, 1960
Genus *Neomediomastus* Hartman, 1969
Genus *Notomastus* M. Sars, 1851
Genus *Paraleiocapitella* M. Sars, 1851
Family Cossuridae Day, 1963
Genus *Cossura* Webster & Benedict, 1887
Family Magelonidae Cunningham & Ramage, 1888
Genus *Magelona* F. Müller, 1858
Family Maldanidae Malmgren, 1867
Genus *Sabaco* Kinberg, 1866
Family Opheliidae Malmgren, 1867
Genus *Ammotrypanella* McIntosh, 1878
Genus *Ophelia* Savigny, 1822
Genus *Ophelina* Örsted, 1843
Genus *Tachytrypane* McIntosh <i>in</i> Jeffreys, 1876
Family Orbiniidae Hartman, 1942
Genus *Califia* Hartman, 1957
Genus *Scoloplos* Blainville, 1828
Family Paraonidae Cerruti, 1909
Genus *Aricidea* Webster, 1879
Genus *Cirrophorus* Ehlers, 1908
Genus *Levinsenia* Mesnil, 1987
Genus *Paradoneis* Hartman, 1965
Genus *Paraonides* Cerruti, 1909
Family Scalibregmatidae Malmgren, 1867
Genus *Asclerocheilus* Ashworth, 1901
Genus *Pseudoscalibregma* Ashworth, 1901
Family Travisiidae Hartmann-Schröder, 1971
Genus *Travisia* Johnston, 1840

### 2.5 Stacked bar graphs

**Fig. 3:**
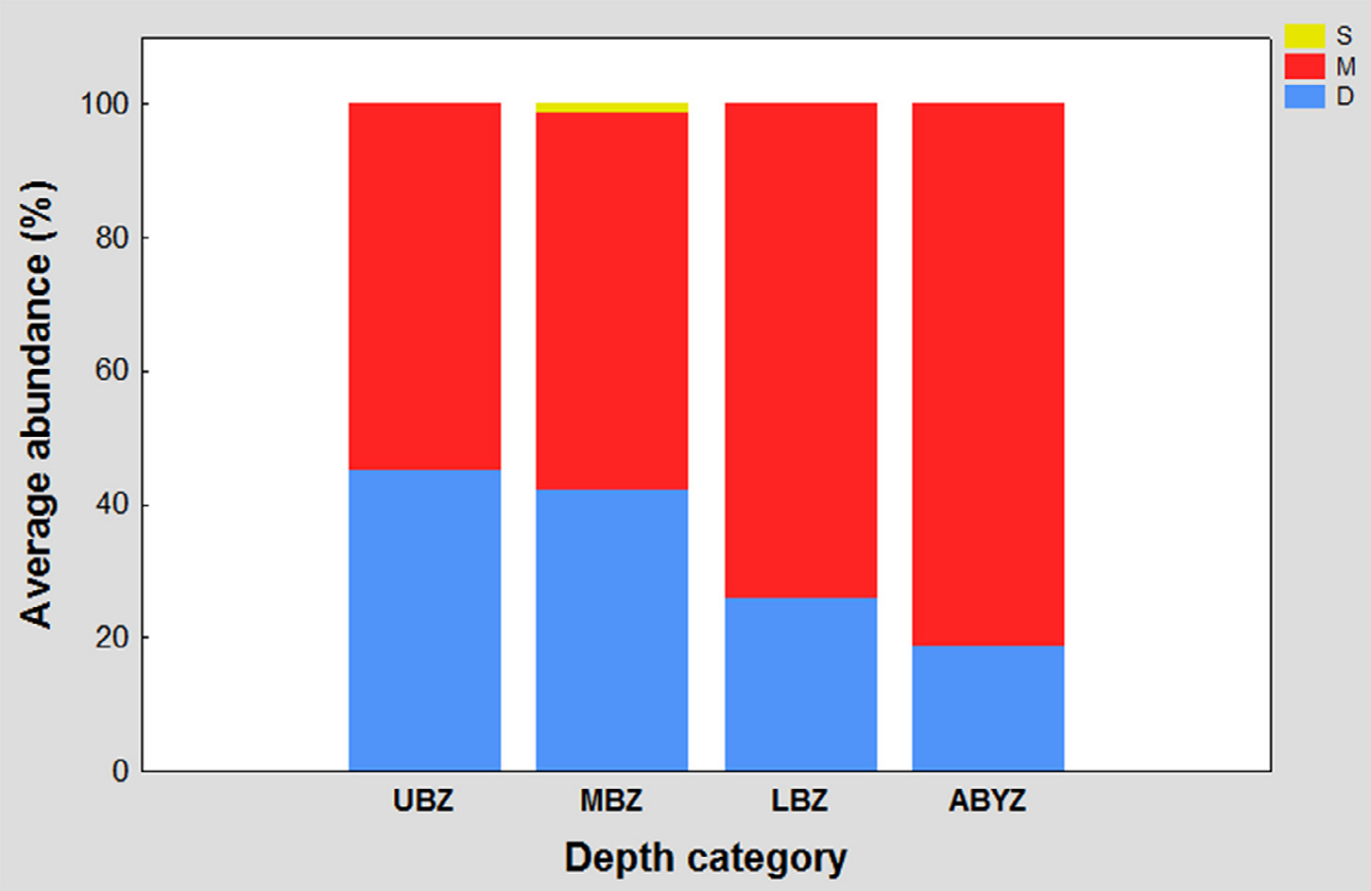
Motility traits percentage contribution to average abundance in each depth category. The letters meaning in the acronym are M = motile; D = discretely motile, and S = sessile. Upper bathyal zone (UBZ); middle bathyal zone (MBZ); lower bathyal zone (LBZ) and abyssal zone (ABYZ).

The assignment of Polychaeta genera to the feeding guilds was carried out following the proposal of Jumars et al. (2015) [12]. The elaboration of the 100% stacked bar graphs, was done based on the feeding guilds, and motility traits average and subsequently the percentage contribution of each biological trait to the depth categories. Based on this relative abundance matrix, the 100% stacked bar graphs were generated using the STATISTICA 7 software, (Figs. 1, 2, 3).

## Ethics Statements

The authors declare that the manuscript adheres to Ethics publishing standards.

## CRediT Author Statement

**Octavio Quintanar-Retama**: Conceptualization, Methodology, Formal analysis, Investigation, Data curation, Writing – original draft, and visualization. **Ana Rosa Vázquez-Bader**: conceptualization, methodology, and writing – Review & editing. **Adolfo Gracia**: Conceptualization, Methodology, Investigation, Resources, Writing – Review & editing, Supervision, Project Administration, and Founding acquisition.

## Declaration of Competing Interest

The authors declare that they have no known competing financial interests or personal relationships that could have appeared to influence the work reported in this paper.

## Data Availability

Polychaete dataset of the southwestern Gulf of Mexico including: taxonomic checklist, abundance, bathymetric distribution, functional diversity, geographic location, and sampling sites depth (Original data) (Mendeley Data).
